# Using an mHealth App to Transition Care of Type 1 Diabetes from Parents to Teens: Protocol for a Pilot Study

**DOI:** 10.2196/10803

**Published:** 2018-10-30

**Authors:** Bree E Holtz, Katharine M Mitchell, Denise D Hershey, Shelia R Cotten, Amanda J Holmstrom, Joshua Richman, Julie K Dunneback, Michael A Wood

**Affiliations:** 1 Department of Advertising and Public Relations Michigan State University East Lansing, MI United States; 2 College of Nursing Michigan State University East Lansing, MI United States; 3 Department of Media and Information Michigan State University East Lansing, MI United States; 4 Department of Communication Michigan State University East Lansing, MI United States; 5 Department of Surgery University of Alabama-Birmingham Birmingham, AL United States; 6 Department of Pediatric Endocrinology Sparrow Health System Lansing, MI United States; 7 Department of Pediatrics University of Michigan Ann Arbor, MI United States

**Keywords:** mHealth, adolescents, type 1 diabetes, mobile phone

## Abstract

**Background:**

Type 1 diabetes mellitus (T1DM) afflicts approximately 154,000 people under the age of 20 in the United States. Most people with T1DM are diagnosed at a young age, and parents have to take on the responsibility of T1DM management. Eventually, the child must begin to transition to self-management. Adolescents often struggle to take on responsibility for all the necessary tasks to successfully self-manage their T1DM. In fact, approximately three-quarters of adolescents are not achieving American Diabetes Association–recommended glycated hemoglobin (HbA_1c_) targets. This lack of adherence can lead to negative health outcomes.

**Objective:**

The goals of this interdisciplinary proposal are as follows: (1) to develop a unique and theory-driven technology using a mobile phone app to promote self-management behaviors for adolescents aged 10-15 years with T1DM and their parents and (2) to explore the feasibility and impact of the self-management mobile app.

**Methods:**

This study has two phases: app development and pilot testing. In the app development phase, the app will be conceptualized and a prototype will be tested. In Phase 2, the mobile app will undergo pilot testing to determine its feasibility and impact on diabetes self-management.

**Results:**

The pilot test was launched in September 2017. Data collection for the final pilot test is underway, and results are forthcoming.

**Conclusions:**

Adolescents with T1DM and their parents can have a difficult time managing the transition of diabetes care. It is hoped that this app can help. The focus groups and prototype testing have indicated promising outcomes of app use.

**Trial Registration:**

ClinicalTrials.gov NCT03436628; https://clinicaltrials.gov/ct2/show/NCT03436628 (Archived by WebCite at http://www.webcitation.org/72tHXTE2Z)

**International Registered Report Identifier (IRRID):**

RR1-10.2196/10803

## Introduction

Type 1 diabetes mellitus (T1DM) afflicts approximately 154,000 people under the age of 20 in the United States [1-3]. Most people with T1DM are diagnosed at a young age, and parents take on most of the responsibility for T1DM management. Optimal treatment requires the entire family to quickly learn about the disease and to oversee its management [4]. Starting in the early teen years, the child must begin to transition to self-management. During this transition, adolescents often struggle to take on responsibility for all the necessary tasks in order to successfully manage their T1DM [2,5]. Approximately three-quarters of adolescents are not achieving American Diabetes Association (ADA)–recommended glycated hemoglobin (HbA_1c_) targets. Achieving these targets requires consistent and dedicated management [6]. A lack of adherence to the prescribed treatment regimen can lead to negative health outcomes, such as the development of diabetic ketoacidosis, increased infections, and hospitalizations [7-9].

One reason for the lack of adherence is that as a child matures into an adolescent, communication between the child and his or her parent becomes increasingly difficult, and conflict often increases. There are data to suggest that improving communication between the adolescents and their parents facilitates a smoother transition to self-care and is one way to improve HbA_1c_ [10]. Mobile health (mHealth) technologies can also ease this transition by facilitating trust-building and improving family communication, relationships, and health outcomes. Nearly three-quarters of American teens (13-17 years old) have access to a smartphone [11]. Our hypothesis is that a mobile phone-based intervention that focuses on improving family communication will be effective in providing both the adolescents and their parents with real-time, tailored information about their diabetes and tools for communicating effectively**.**

Easing transition difficulties in an effort to increase positive health outcomes is imperative to the well-being of adolescents with T1DM and their families, and studies have shown that participants using mobile phone interventions had higher adherence rates and more participation than those that did not use the intervention [12]. Therefore, this study will develop a mobile app that addresses the difficulties of the adolescent transition to self-management and pilot test the app to determine its feasibility and impact on diabetes self-management. Below, we discuss the methodology for developing the app as well as the theoretical foundations guiding this development.

## Methods

### Study Design

This study has two phases that correspond with the study aims (Textbox 1): app development and pilot testing. In the app development phase, the app will be conceptualized and a prototype will be tested. In Phase 2, the mobile app will undergo pilot testing to determine its feasibility and impact on diabetes self-management. We did not utilize a control group because this is a feasibility study [13].

### Theoretical Foundation

**Figure 1:**
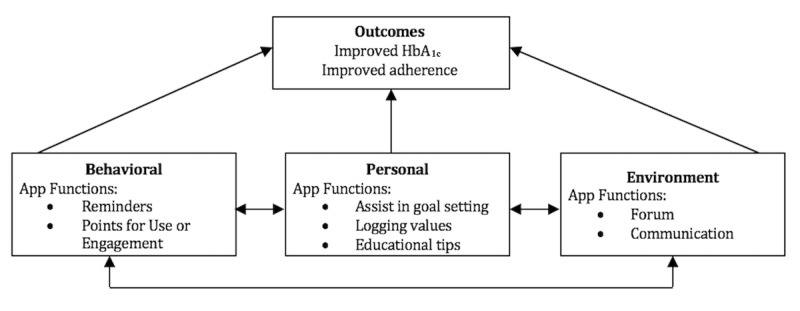
Theoretical model. HbA_1c_: glycated hemoglobin.

The social cognitive theory will inform the development of the intervention. The use of this theory to guide interventions has demonstrated success in changing behaviors around disease management [14-17]. The social cognitive theory posits that a change in an individual’s environmental, personal, or behavioral factors will impact the other two factors [18-21]; thus, the relationship among these factors is reciprocal. The self-management mobile app is being developed to lead to changes in each of the three factors. For example, to change the adolescents’ environmental factors, we will provide social support through a forum feature [22-24]. We believe that receiving messages from their parents via the self-management mobile app will also be a part of the environmental change [25-28]. Personal factors will be modified by prompts sent to the adolescents to set small and achievable daily goals, blood sugar reminders, and educational tips about living with T1DM to improve the adolescents’ self-efficacy [29-34]. Behavioral change will result from reinforcement through reminders and awarding points for achieving goals and entering data. The points encourage use (behavior), which we predict will improve adolescent adherence [35]. As a result of all of these factors, children will have an improved HbA_1c_, a higher quality of life, and reduced conflict with their parents, which we believe will reinforce the use of the self-management mobile app, strengthening the links. Figure 1 shows the theoretical model for the intervention.

**Specific aims.**

**Phase 1**

*Specific Aim 1: Develop a type 1 diabetes self-management mobile phone app intervention for parents and adolescents*

- Objective 1: Develop the self-management mobile app and finalize content
- Objective 2: Prototype testing

**Phase 2**

*Specific Aim 2: Conduct pilot testing of the self-management mobile app to determine its feasibility and impact on diabetes self-management*

- H1 (primary outcomes): Measures of app usage will be associated with increased adherence to self-management and lower glycosylated hemoglobin.
- H2 (secondary outcomes): Measures of app usage will be associated with increased quality of life, self-efficacy, and social support and decreased conflict between the adolescents and their parents.

### Phase 1: Technical App Development

The technical app development (Phase 1) will take place over a 9-month period. This process will be monitored through set goals, and progress will be measured by achieving two objectives of developing the self-management mobile app and finalizing content. To complete these objectives, content development will be completed based on several sources, including *Understanding Diabetes* [36] and other literature from the ADA, in conjunction with an expert panel who will also advise on the project during implementation. This panel includes a pediatric endocrinologist, a pediatric diabetes nurse practitioner, a college student with T1DM, and a parent of an adolescent with T1DM (not part of the study).

Based upon best practices in app design principles [37], we will also conduct focus groups with participants recruited from our target population. We will invite 6 adolescents and their parents for concept-testing focus groups. We will present the app concept and mock screenshots to the participants and solicit feedback with regard to the app’s look and feel, message wording, and what would help the adolescents to log their values. The data from the focus groups will inform message content refinement, the timing of the messages, their appropriateness, and the ease of logging information. This information will inform the app’s technical development. The technology development (app interfaces, Web portal, and servers) will be informed by the focus groups, concept testing, and best practices literature. We have 10 benchmarks with deadlines for app development (Table 1).

Once the prototype is developed, the objective 2 will be met by prototype testing of the app. We will recruit 10 family dyads (each dyad includes a teen and his or her parent, n=20) to use the self-management mobile app prototype over a 4-week period. We will conduct usability interviews, measuring perceptions and satisfaction with the app. Additionally, server information will be used to quantify learning time, the efficiency of use, engagement, and user errors. All of the problems and issues identified in the usability interviews will inform app development and refinement.

#### The Mobile App

##### App Functionalities

The self-management mobile app will be an intervention mechanism to encourage adolescents aged 10 to 15 years to self-manage their diabetes by recording their values while allowing their parents seamless and unobtrusive access to the same blood glucose (BG) values, carbohydrate intake, and physical activity data, which will be time-stamped, encrypted, and sent to a remote server for storage.

**Table 1:** App development benchmarks.

Step	Time (in days)
Server management	13
User management	13
Server side logic	12
Data integration	13
Push logic	6
Versioning	12
Cache logic	6
Synchronization	8
Wireframe development	8
User interface design & development	33

The parent users will have a separate log-in and will be able to review their child's history of BG values, carbohydrate intake, and physical activity from their own phone to help their child make decisions about managing their diabetes. Parents may also send messages via the app to their child. However, the parent can still communicate outside of the app, and we will ask about this communication in the poststudy interviews.

Along with customizable test reminders and BG ranges, the app will also have an educational component and a moderated forum for the adolescents and the parents (separate forums) to post and discuss questions and comments to peers. The adolescents will be incentivized through the use of points accumulated based on usage. These points can be used by the adolescents to purchase accessories for a superhero avatar. As this is a feasibility study, we will have the adolescents manually input their BG readings into the app. While this may seem cumbersome, most meters and pumps do not have the ability to automatically send data through Bluetooth. Once we can determine the feasibility and other design requirements through this pilot, we will develop the app to be Bluetooth-enabled. The participants and their parents are responsible for notifying their medical providers regarding any issues with their diabetes. Our app will not cause an actual health concern, as it is just a monitoring and communication device.

##### Technical Details of the App.

The mobile app will be initially developed for the Android platform. Future iterations will include an iOS platform. Focusing on one operating system will allow us to develop a self-management mobile app using the highest-quality control standards. We have selected Android because it is the world’s most popular platform [38]. Mobile phones will be provided to any participant who does not have a phone or one compatible with the app. The app will feature separate and secure log-in mechanisms for the adolescent user and parent user. All data will be stored on a remote encrypted server rather than the phone itself. If the phone is lost or stolen, we will have the ability to remotely erase the phone’s contents and turn off the data plan associated with it. Technical specifications and functions of the app have been presented in Textboxes 2 and 3.

### Phase 2: Intervention Study Methods

This portion of the intervention involves having the adolescents and their parents use the app over the course of 3 months.

#### Study Setting

Recruitment will take place in the Sparrow Hospital’s Pediatric Subspecialty Clinic in Lansing, Michigan, United States. The Sparrow Endocrinology Clinic team consists of 1 registered pediatric dietitian, 1 full-time nurse practitioner, 2 certified diabetes educators, and 2 endocrinologists, all of whom provide diabetes care 4 days per week within the larger subspecialty clinic. All patients are scheduled to come to the clinic every 3 months for care.

#### Sample and Recruitment of Participants.

The clinic nurse diabetes educator will act as the site coordinator. Participants for the pilot testing will be recruited from the hospital’s Pediatric Endocrinology Clinic. Invitations will be mailed three times over 2 months to all of the potential participant dyads, as identified by hospital providers. Additionally, the health care providers in the clinics will be given a presentation on the study by the researchers so they can offer it to the families who meet the inclusion criteria during any typical interactions they have with the adolescent and parent.

**Technical specifications.**

- Operating system: Android
- Data storage: Remote encrypted server
- Log-in mechanisms: Separate and secure log-in mechanisms for the adolescent user and parent user

**App functions. BG: blood glucose.**

**Child functions:**

- Incentivized through the use of points accumulated based on usage
- Send messages via the app to their parent
- Customizable test reminders and BG ranges
- An educational component
- A moderated forum for the adolescents and the parents (separate forums) to post and discuss questions and comments to peers

**Parent functions:**

- Review their child’s history of BG values, carbohydrate intake, and physical activity from their own phone
- Send messages via the app to their child
- Customizable test reminders and BG ranges
- An educational component
- A moderated forum for the adolescents and the parents (separate forums) to post and discuss questions and comments to peers

**Inclusion criteria.**

**Adolescents must:**

- Have a type 1 diabetes mellitus (T1DM) diagnosis according to the American Diabetes Association practice guidelines [34]
- Be 10-15 years old
- Have had a diagnosis of T1DM for at least 6 months; have glycated hemoglobin>7 [35]
- Have had at least two outpatient visits in the past 2 years
- Be treated at the local clinic for diabetes
- Be fluent in English
- Have a parent or guardian willing to participate
- Be allowed to use a mobile phone for the study
- Have permission from their care team

**Parents must:**

- Have an adolescent with T1DM who is 10-15 years old
- Be fluent in English
- And have daily access to email and the internet (for appointment reminders and technical support)

**Exclusion criteria.**

**Adolescents:**

- With a diagnosis of a major psychiatric or neurocognitive disorder (eg, traumatic brain injury, dementia, schizophrenia, bipolar disorder, borderline personality disorder, and intellectual disability)
- With significant medical conditions other than type 1 diabetes
- Being treated for thyroid disorders, celiac disease, or eating disorders
- In foster care

**Parents:**

- With a diagnosis of a major psychiatric or neurocognitive disorder (eg, traumatic brain injury, dementia, schizophrenia, bipolar disorder, borderline personality disorder, and intellectual disability)

If interested, the parent will contact the researchers to learn more about the study. A researcher will be in the waiting room of the pediatric endocrinologists, providing study information to possible participants. The researcher will explain the study and determine if the adolescent and parent are eligible through a brief screening questionnaire. We will recruit a total of 70 adolescent and parent dyads, which would correspond to similar past research studying T1DM and mobile phone interventions [12]. Inclusion and exclusion criteria have been presented in Textboxes 4 and 5, respectively.

#### Study Procedures.

During the study, there will be 4 contact points with the participants: the enrollment or baseline data collection visit (Visit 1), a midpoint review (Visit 2, 3 months after Visit 1), an end-of-study visit (Visit 3, 6 months after Visit 1), and a follow-up visit 3 months after app usage has stopped (Visit 4). Visit 1 (month 0) will be scheduled during the screening process. At Visit 1, a member of the research team will review and obtain informed consent (parent) and assent (adolescent) from the participants. The participants will then complete the baseline surveys to measure self-efficacy, quality of life, and social support and conflict; the laboratory will test the teens’ HbA_1c_, and we will download data from their glucometer or pump. If a participant does not have a mobile device that can download and operate the app, the research team will provide the participant with a device and data plan for the duration of the study. The researcher will demonstrate how to use the technology and have the participants use each of the functions. At Visit 2 (month 3), a research team member will test the adolescents’ HbA_1c_ and download data from their glucometer or pump. The participants will also complete the survey at the midpoint. The midpoint data will be used to demonstrate whether any change at 6 months is part of an increasing trend, a plateau, or a waning trend. At Visit 3 (month 6), the participants will complete the survey and participate in interviews. Additionally, the adolescents’ HbA_1c_ will be tested, and the meter or pump readings will be collected. Finally, any participant that has borrowed any equipment will need to return it at this time. Qualitative methods will also be used to better examine perceptions and intentions, whether participants were light users, or why participants dropped out of the study [39]. After Visit 3, the participants will not use the app for 3 months. At Visit 4 (month 9), all the participants will complete the survey. The researchers will test the adolescents’ HbA_1c_ and download data from their glucometer or pump.

#### Outcome Measures

Specifically, the aim 2 seeks to determine the feasibility and impact on diabetes of the self-management mobile app among the adolescents and their parents. Demographic information will also be collected including current age, age at diagnosis, race, gender, parental status (ie, mom, dad, stepdad, etc), and method of diabetes treatment (eg, pump).

Hypothesis 1 seeks to demonstrate that adherence to self-management correlates with mobile app usage and a decrease in HbA_1c_. HbA_1c_ data will be collected through the hospital laboratory system. The patient will be provided with a laboratory slip to obtain the baseline HbA_1c_ value. The patient will be instructed to obtain the lab results within 1 week. Once the results are obtained, they will be faxed to the principal investigator via a secure fax line. Adherence will be measured in two ways. First, the participants will complete the Diabetes Behavior Rating Scale [40]. Second, we will use data from the server to determine if they are monitoring blood sugar on schedule and what mechanisms of the app helped the adolescents follow the medical recommendations. For example, we will use time-stamped log-ins, number of sections the user visited, number of individual logs, communication through the app, educational information accessed, and duration of use per log-on.

Hypothesis 2 states that through use of the app, the participants’ self-efficacy [29,41,42], quality of life [43-45], and perceptions of social support [46-48] will improve, and conflict [49] between the child and parent will be reduced from baseline to 3 months. These secondary outcomes are key in helping the adolescents maintain adherence and a normal HbA_1c_ [50-52] and will be measured through surveys. The survey instruments, the participants who will complete them, and reliability for each measure can be found in Table 2.

#### Analysis and Statistical Power

This is a feasibility study, so all analyses will be exploratory, focusing on identifying temporal trends in the self-management mobile app usage and changes in study outcomes and identifying associations between outcomes. Preceding the analyses, we will calculate summary statistics for all measures as well as exploratory plots (histograms and box-plots) for all measures at each time point to identify outliers and potentially spurious values.

Trends over the 4 visits will be examined graphically as a function of time using nonparametrically smoothed plots to help identify when potential effects reach their maximum, plateau, and begin to decrease, which may indicate a time to target for additional interventions. At each time point, we will calculate pairwise correlations between the self-management mobile app usage measures and all study outcomes and examine bivariate scatter-plots with estimated trends for each pair. Distribution-appropriate correlations (Pearson or Spearman) will be calculated as appropriate for each measure and formally tested for statistical significance. These analyses will also be repeated using change from baseline as the outcome rather than the score itself, both as a simple bivariate analysis and using linear regression to model the change as a function of the app usage measures while controlling for baseline values. Analyses will also be repeated using paired *t* tests for differences in HbA_1c_ values from baseline to posttest to measure self-management adherence.

Statistical power will be established based on a small pilot study. A 2010 report on a small randomized controlled trial (RCT) of an mHealth intervention for T1DM in teenagers showed a significant change in self-management adherence from a mean of 3.7 (SD 0.4) at baseline to 3.9 (SD 0.4) at the 12-week follow-up, at an effect size of *d*=0.64 [53]. Given our longer follow-up period (6 months), we expect to observe a larger effect size in our feasibility study because HbA_1c_ has low variability in 3 months [54]. While using a small pilot study to calculate power may not be ideal, it provides a foundation for us to approximate this study’s sample size in order to estimate the critical parameters necessary for this study [55]. The 2010 study also reported no change in HbA_1c_ for the intervention group, but there was an increase in the control group, with SDs ranging from 1.2 to 1.9. Considering that their groups were imbalanced with respect to HbA_1c_ at baseline, it is difficult to project a difference for our study except to note that observing no change in HbA_1c_ may be an improvement over a possible increase in adolescents with usual care. Despite our limited sample size, assuming the use of two-sided tests with a type 1 error rate of 0.05, with 70 participants we will have 80% power to detect relatively modest correlations of .29.

**Table 2:** Survey instruments.

Outcome	Measure	Reliability (α)	Participant
Adherence	Diabetes Behavior Rating Scale	.86	Child or parent
Glycated hemoglobin	Blood test—HbA_1c_	N/A^a^	Child
Social support	Multidimensional Scale of Perceived Social Support	.81	Child or parent
Social support	Diabetes Family Behavior	.79	Child or parent
Self-efficacy	Diabetes Empowerment Scale-Short Form	.84	Child
Quality of life	Pediatric Quality of Life Inventory (general & diabetes specific)	.88	Child
Conflict	Diabetes Family Conflict Scale	.91	Child or parent

^a^N/A: not applicable.

In the event of unanticipated difficulties with recruitment or of attrition of up to 20%, leaving a final sample of 56, the correlation detectable with 80% power increases only slightly to .32. For *t* tests, N=70 provides 80% power to detect an effect size of 0.30, which is smaller than that reported in the RCT, while the effect size detectable with N=56 increases slightly to 0.34 [53].

Additionally, the primary foci of this study include assessment of the following: (1) the effect size of the intervention to power a larger study; (2) feasibility of the app; (3) practicality of data collection procedures; and (4) the ability to implement. We will also perform a post hoc analysis on personal characteristics and satisfaction related to use of the system. At the completion of the study (month 9), we will ask both those who completed the whole intervention and those who withdrew early about their experience with the intervention, including what worked well and what did not. Additionally, we will use the IBM computer usability satisfaction questionnaire to assess usability [56].

The interview data will be analyzed by developing broad code categories based upon perception themes and will serve as a preliminary sorting tool. The researchers will then use thematic analysis and create a list of common perceptions. Once a coding scheme is developed, two coders will perform a pretest by coding randomly selected interview transcripts in order to measure reliability. Coders will work together to reconcile any disagreements; this will allow us to achieve a high level of reliability.

## Results

The study has been approved by the Institutional Review Board of Michigan State University, United States. We conducted focus groups prior to app development in April 2016 [57]. Prototype testing of the app was conducted in February 2017, and it included feedback regarding usability and satisfaction of the app [58]. The final pilot test was launched in September 2017. Data collection is underway, and results are forthcoming.

## Discussion

Adolescents with T1DM and their parents often struggle during the transition from parent care to adolescent self-management [59,60]. This transition to young adulthood is challenging for all families, but families facing the added complexity of diabetes management often find this time especially challenging. The proposed intervention is innovative as it aims to shift diabetes management responsibility to the adolescent and increase their independence while encouraging parents to remain engaged and supportive of their adolescent in an effort to ease some difficulties of this challenging time.

To do this, a mobile app for adolescents and their parents is proposed. Previous mHealth research indicates that mobile apps provide an innovative approach to impact health [12,61] using technology that is frequently part of adolescents’ and parents’ daily life [62,63]. However, very few existing apps have shown significant or sustainable improvements in adolescent self-management and overall health outcomes,[64] and none have directly involved the parents in the use of the app. In some apps that are currently available, the parents can receive the BG value, but they are not given any prompts on how to positively address blood sugar results and plan for care with their child. Our app will include communication through the app that may allow for adolescents to ask for help and communicate more honestly with their parent than they would in face-to-face communication [65]. This improved communication between adolescents and their parents has been shown to facilitate a smoother transition to self-care by improving communication between parents and adolescents, providing adolescents with tangible self-management skills, and providing families with social support and has been shown to improve HbA_1c_ [10,66,67].

One difficulty of using mobile app technology is keeping adolescents engaged with app use over long periods of time. This is also an issue when designing an intervention with outcomes that take 3 or more months to realize significant changes. Data suggests that the majority of user engagement begins to drop after 21 days of use [68,69]. To combat this difficulty, the app includes a points-based customizable avatar. However, it is important to note that researchers anticipate seeing a similar trend in engagement. Another difficulty seen in many studies is problems with recruitment and retention. To combat these difficulties, participants will be contacted frequently, flexible scheduling will be used for participant study meetings, and study newsletters will be sent to participants. In the event of unanticipated difficulties with recruitment or attrition, a final sample of 56 is acceptable [53].

After we have successfully completed this study, the data gathered will be integrated with machine learning in order to modify the app for a wider audience and better tailor the app to the individual user’s behavior and usage preferences. Once completed, we will design and conduct a full-scale RCT. We will also expand the project to iOS (Apple phones). Additionally, we will seek to integrate the data into the patient’s electronic health record.

## Appendix

Multimedia Appendix 1 Peer-reviewer report from the American Diabetes Association's Research Program.

## Abbreviations

ADA: American Diabetes Association
BG: blood glucose
HbA_1c_: glycated hemoglobin
mHealth: mobile health
RCT: randomized controlled trial
T1DM: type 1 diabetes mellitus
