# Supplementary material for: Using an mHealth App to Transition Care of Type 1 Diabetes from Parents to Teens: Protocol for a Pilot Study
Source: JMIR Res Protoc. 2018 Oct 30;7(10):e10803. doi: 10.2196/10803 (PMC6234347; doi:10.2196/10803)
Supplement: Multimedia Appendix 1 [file resprot_v7i10e10803_app1.pdf]

American Diabetes Association.
RESEARCH **PROGRAMS**

Dear Dr. Holtz:

Thank you for your interest in the American Diabetes Association's Research Program. Earlier today we sent an email informing you that we intend to fund your Innovative Clinical or Translational Science Award application #1-16-ICTS-045, titled *Using an mHealth app to transition care of type-1 diabetes from parents to teens*. As follow up to that notification, please see below for the reviewers' final review comments and a summary of the discussion around your grant:

Reviewer 1:

A) RESEARCH APPROACH

This application seeks to develop an app to facilitate transition in self-management from parents to children (10-15y) by enhancing communication, providing a neutral channel for transmission of data about self-care behaviors and reminders between parents and teens, and also providing a means for social support and behavior modification. The app will be developed using focus group results indicating what components the app needs to contain to make it usable by youth and their parents, is driven by the Social Cognitive Theory and will be beta tested for feasibility, with the possibility of adaptation before full RCT role out. Primary outcomes are increased adherence to self-management behaviors and improved A1c for the youth.

There are some minor concerns about whether youth will be accurate in their entries of self-care behaviors (or lack thereof), though downloads of management devices will be a concrete test of validity; and whether they will use this app in a sustained fashion as the app as conceptualized appears intrusive to use. This will be tested however and does not diminish enthusiasm but has significant implications for prolonged use of the app. Other concerns regard the lack of inclusion of health care providers regarding receipt of information, specifically when glucose ranges outside the normal range and the adolescent does not take corrective action. The application states that the app is just a monitoring and communication device. There is some concern regarding recruitment goals both regarding achieving a 50% goal from available families (150, need 70) and the representative nature of the sample obtained for feasibility testing. Also in this regard, the sample should be balanced regarding pump va non-pump users, as it is well documented that pump families are a "select" population, with greater resources, educational attainment and enhanced use of technology. This is somewhat offset by the pilot study being conducted in a single setting so that medical care is being monitored and changes in medical management accounted for, and the investigators specifically couching this application as the development and feasibility of use phase. Mid point and hard outcome assessments are considered a strength to monitor trends in use.

B) SIGNIFICANCE FOR PEOPLE WITH DIABETES

Transition to self-management in youth has long been a problematic issue in diabetes care and has recently received a great deal of attention, particularly in the development of comprehensive programs to facilitate this outcome. This stand alone app could be quite helpful and efficient in helping to accomplish these goals: successful transition in care from parent to child, improving adherence and better glycemic control. If this study is successful, it will lay the ground work for a fully powered RCT which can test effectiveness regarding significant A1c reduction and sustainability of use.

C) INNOVATION

Use of apps to improve adherence is not innovative, but the proposed components target essential elements which are known barriers to transitioning care and link parents and children. Use of theoretically

driven components which have previously predicted behavior change suggest a somewhat novel approach to the development of an adherence app.

D) INVESTIGATOR/ENVIRONMENT

The team represents the needed expertise and seems to have been working together on this effort for the past year. There needs to be mention of contingency plans regarding changes in treatment personnel.

E) FACILITIES

The families will be recruited from a single treatment site: Sparrow Hospital's Pediatric Subspecialty Clinic. There is concern that a 50% recruitment rate would be ambitious and no other fallback plan is presented. There are no discriptors presented regarding what percentage of their population has an A1c above 7.0 in the appropriate age range. Also, demographics of the sample as well as percentages of pump non-pump users would be important in understanding whether this app would be broadly acceptable among families with teens with type 1.

F) HUMAN SUBJECTS/ANIMAL WELFARE

Human subjects concerns are not adequately addressed. Consenting issues are not addressed, particularly with regard to assent of minors. Also, the issue of adverse outcomes is not addressed. This regards transmission of medical information to care providers as well as flagging for corrective action if a glycemic level is outside the normal range in either direction. These issues need to be addressed before this protocol is implemented, even as this application regards development and feasibility testing.

G) BUDGET/CTSA (IF APPLICABLE)

No budgetary concerns.

H) OTHER COMMENTS AND/OR AREAS OF CONCERN

There is significant enthusiasm for this application but there are recruitment, human subjects and treatment concerns which could be easily fixed before implementation.

Reviewer 2:

A) RESEARCH APPROACH

Strengths

- The application is well-written and straightforward

- Iterative approach to development of the mHealth tool is a major strength

- Inclusion of preliminary data regarding acceptability from the end-users (parents and adolescents) also a strength

- The proposed intervention in novel

- Theory is incorporated into the development and evaluation phase

- Builds on content from previously developed programs (understanding diabetes, family teamwork intervention, ADA)

- Engages an expert panel with pediatric endocrinologists, nurses, college student and parents of adolescents with type 1 DM

- The scope of work and sample size are appropriate for a pilot study

- Plan to start with android platform initially is well justified

- Strong primary and secondary outcomes

Weaknesses

- Design could be strengthened by the inclusion of a control group though it is not necessary

- There is some concern about the feasibility to reach recruitment goal

B) SIGNIFICANCE FOR PEOPLE WITH DIABETES

- Transition for adolescents with type 1-diabetes from management with the help of parents to self-management represents a period of risk for the adolescents and is an important area in need of interventions.

- Use of technology based intervention will likely resonate with teens.

C) INNOVATION

- Using technology to link parents and teens is an innovative approach with good face validity.

- The use of an app to assist with self-management is rapidly becoming less novel, though fewer are evidence based thus the proposed study could help address that evidence gap.

D) INVESTIGATOR/ENVIRONMENT

Very strong investigative team with necessary expertise to complete the proposed study

E) BUDGET/CTSA (IF APPLICABLE)

Budget appears appropriate for the given scope of work

F) OTHER COMMENTS AND/OR AREAS OF CONCERN

N/A

Reviewer 3:

A) RESEARCH APPROACH

The focus of this proposal is development of a novel diabetes application, with several novel features including linking child self-monitoring data to a parent in order to facilitate better family communication and oversight in a non-confrontational manner. Development and testing of the proposed application is structured into two aims: Aim 1: To develop/finalize the diabetes self-management mobile phone app and test the prototype Aim 2: To conduct a pilot trial with the self-management app to determine feasibility and impact on: (1) adherence and A1c; and (2) quality-of-life. For this second aim, 70 parent-child dyads will be recruited from the Sparrow Endocrinology Clinic. I2U2 usage will be correlated with the endpoints.

Strengths include:

• Clear rationale for components of App, supported by social-cognitive theory and based on a pilot study (focus groups including children, parents, and diabetes educators)

• Diverse App components, which in addition to a standard logging and goals feature, include social support forum, behavioral reinforcement (points and reminders), and non-confrontational communication between parent and child via automatic data sharing with parent

• A clear plan is laid out for App development. Step 1 includes refinement /development of current app

using: (1) medical literature and family teamwork models; (2) an expert panel (includes diabetologist, CDE, college student with DM, parent); and (3) focus groups to solicit feedback on app features and screen shots. Benchmarks and timelines are identified. Step 2 is a 4 week trial of the prototype, to identify and fix technical problems. This will occur over the first 9 months.

• Investigator details a very clear and robust plan for patient retention.

• Proposal includes next steps, noting primary foci of current study are to determine effect size for powering future RCT; determining feasibility of app, data collection procedures, and implementation. Next step plans are to seek R01 funding for RCT.

• Targets an age group in which transition to independence is occurring and thus is likely to benefit from App features

• This is a strong investigative team with complementary expertise necessary to both design the App and successfully implement and study the App. The team includes expertise in health informatics, App development, and clinical diabetes as well as intrafamilial communication. The PI (Holtz) is a relatively young investigator but with a strong track record and the necessary expertise in health informatics to conduct the study, as well as an established collaborative relationship with the other team members

Potential weakness include:

• The first iteration of the App will be designed for Android only. Scientifically this is a valid and logical approach. Although phones are provided for non-Android users, I am concerned that this may still deter participation of iOS users (though conversely, may be an incentive for others who do not current have a smart phone device).

• The proposal seeks to recruit 70 child-parent dyads out of about 150 eligible, and so assumes a nearly 50% participation rate, over a 3-6 month enrollment period. Because of the non-invasive nature of the study, and appeal of new technology/Apps, this may be an achievable goal but investigators do not detail what steps could be taken if recruitment goals are not reached with current study plan and inclusion/exclusion criteria.

• Not having a control group is appropriate for an initial pilot study, though may see improvements in endpoints of adherence, A1c, and QOL due to trial participation. Correlating app useage with these outcomes is logical and appears to avoid some of this potential bias arising from trial participation.

B) SIGNIFICANCE FOR PEOPLE WITH DIABETES

While there are many Apps now available for patients with diabetes, few are developed with a robust evidenced-based approach. This proposal aims to develop a diabetes app based on social cognitive theory and interfamilial relations in diabetes, and will prospectively study the impact of the App. A new, technology-based approach that aids in maintaining adherence and glycemic control during the transition to independence that occurs in adolescence in T1D is an area with potential high impact, as this is an area of high need and known poor control.

C) INNOVATION

Innovative in evidence based approach to app development, and inclusion of unique app features, particularly in facilitating non-confrontational communication between independent adolescent and parent and a social forum.

D) OTHER COMMENTS AND/OR AREAS OF CONCERN

None

Reviewer Discussion Summary:

OVERALL SUMMARY AND COMMENTS

There was considerable enthusiasm for this application which describes the development process and preliminary clinical efficacy testing of an app to help transition in care to teens from parents. Reviewers appreciated that construction of the app is based upon operationalizing proven constructs of the Social Cognitive Theory that have been shown to improve healthful behaviors, and based on focus group review of components. Reviewers also evaluated strengths as the ability of this format to reduce parent-child conflict by submission of self-management data directly into the app rather than from child to parent; helping the youth to take corrective actions when glucose is out of range; and the ability to validate youth self-report through input of data from downloads and health records. The research team was deemed to have the needed expertise to carry out the project, with the exception of the addition of a behavioral psychologist familiar with the diabetes care regimen. Concerns centered around recruitment feasibility, the need to stratify the testing sample regarding use of other technologies, and the lack of inclusion of health care providers in receipt of self-management data. The later was noted as both a clinical limitation of the application and as a human subjects concern. The later issue regards incidents when a youth's glycemic values are out of range, no corrective action is taken, and as currently written, a care provider is not contacted.

On behalf of the American Diabetes Association, congratulations on the outcome of your grant application.

Regards,

Katherine Will
Manager, Research Programs
